# MALDI-TOF MS Identification and Clustering Applied to *Enterobacter* Species in Nosocomial Setting

**DOI:** 10.3389/fmicb.2018.01885

**Published:** 2018-08-14

**Authors:** Lucia De Florio, Elisabetta Riva, Annalisa Giona, Etleva Dedej, Marta Fogolari, Eleonora Cella, Silvia Spoto, Alessia Lai, Gianguglielmo Zehender, Massimo Ciccozzi, Silvia Angeletti

**Affiliations:** ^1^Unit of Clinical Laboratory Science, University Campus Bio-Medico of Rome, Rome, Italy; ^2^Unit of Virology, University Campus Bio-Medico of Rome, Rome, Italy; ^3^Unit of Medical Statistics and Molecular Epidemiology, University Campus Bio-Medico of Rome, Rome, Italy; ^4^Internal Medicine Department, University Campus Bio-Medico of Rome, Rome, Italy; ^5^“L. Sacco” Department of Biomedical and Clinical Sciences, University of Milan, Milan, Italy

**Keywords:** MALDI-TOF mass spectrometry, *Enterobacter*, resistance, nosocomial infection, clustering analysis

## Abstract

*Enterobacter* microorganisms cause important bacterial infections in humans. Recently, carbapenem resistant isolates carrying the *bla*KPC gene were described and their clonal transmission in different nosocomial outbreaks reported. In this study, the relative numbers of *Enterobacter* species, their antimicrobial susceptibility along 3 years of observation and the identification ability of the two most common MALDI-TOF platforms were evaluated. A clustering analysis was performed to identify changes in the microbial population within the nosocomial environment. *Enterobacter* were identified using two platforms (MALDI-TOF Biotyper and VITEK MS). Antimicrobial susceptibility was tested by Vitek2 Compact and MIC_50_ and MIC_90_ was evaluated using GraphPad software. Clustering analysis was performed by MALDI-TOF and a dendrogram was built with both platforms and compared. The most frequent species isolated were *Enterobacter cloacae* and *Enterobacter aerogenes* with a gradual increase of *Enterobacter asburiae* in 2017. MALDI-TOF platforms showed a very good sensitivity and specificity except for *E. asburiae* identification that was reliable only by MALDI-TOF MS Biotyper. An increase of resistance for *Enterobacter*, confirmed by the isolation of extended spectrum beta-lactamase (ESBL) strains and the emergence of *E. cloacae* multidrug-resistant (MDR) and carbapenem resistant strains, was observed. A clonal route of transmission involving general surgery and geriatric wards was evidenced as previously described for *Klebsiella pneumoniae* MDR strains in the same nosocomial setting. These data represent an important source of information about the spreading of *Enterobacter* in the nosocomial environment.

## Introduction

Enterobacter species are gram-negative bacilli belonging to the family of Enterobacteriaceae and to the “ESKAPE” pathogens including Enterococcus faecium, Staphylococcus aureus, Klebsiella pneumoniae, Acinetobacter baumannii, Pseudomonas aeruginosa, and Enterobacter species. This group of pathogens is the cause of important bacterial infections in humans (Boucher et al., 2009; Rice, 2010). Enterobacter cloacae and Enterobacter aerogenes represent the most common Enterobacter species, even if other species such as Enterobacter asburiae have been identified as emergent pathogens causing severe infections (Brenner et al., 1986; Stewart and Quirk, 2001; Koth et al., 2012).

*Enterobacter* are intrinsically resistant to some antimicrobials, ampicillin and I and II generations cephalosporins, showing high ability to acquire resistance to most recent drugs, as cephalosporins of III and IV generations, carbapenems and aminoglycoside (Muytjens and van der Ros-van de Repe, 1986; Leibovici et al., 1992). Recently, an increasing prevalence of multidrug-resistant (MDR) *Enterobacter* strains has been observed and in many cases nosocomial strains acquired carbapenemases as *bla*NDM-1 and *bla*KPC limiting the availability of appropriate antimicrobial treatments (Pottumarthy et al., 2003; Deshpande et al., 2006; Blanco et al., 2013). Moreover, these strains are frequently isolated in course of bloodstream infections and severe local infection as pneumonia, especially in critically ill patients (Fridkin, 2001; Marchaim et al., 2008; Castanheira et al., 2011). Given the presence of these two concomitant important factors, this group of pathogens is progressively receiving more attention in clinical practice for the challenge it poses today both to public health and to the management of the spread of infections within hospital environments (Hargreaves et al., 2015; Sidjabat et al., 2015; Zeng et al., 2016). The first *Enterobacter* strain carrying *bla*KPC gene was described in a septic patient in the year 2001 (Hossain et al., 2004). Since this first identification, sporadic cases as well as several outbreaks caused by resistant *Enterobacter* strains have been described worldwide (Bratu et al., 2005; Haraoui et al., 2013; Kiedrowski et al., 2014; Gomez-Simmonds et al., 2016). In recent surveillance studies on carbapenem resistant gram-negative strains, the isolates were represented primarily by *K. pneumoniae* and secondly by *Enterobacter* species carrying the *bla*KPC gene (Landman et al., 2011). The clonal dissemination of carbapenem resistant *Enterobacter* has been described in different nosocomial outbreaks, raising interest in the molecular epidemiology of these strains (Marchaim et al., 2011; Haraoui et al., 2013; Markovska et al., 2014; Villa et al., 2014). In a recent study, whole-genome sequencing was applied to *Enterobacter* clinical isolates from the United States, South America, and the Mediterranean region with the aim to increase knowledge on the genetic characteristics of these emerging pathogens. The study revealed that these strains, evolving from a unique ancestor, follow a clonal spread and that plasmids harboring *bla*KPC gene were horizontally transferred between strains (Chavda et al., 2016).

In this study the relative numbers of *Enterobacter* species and the antimicrobial susceptibility of the strains isolated along 3 years of observation from 2015 to 2017 were evaluated. Moreover, the identification ability of the two most common MALDI-TOF platforms used in clinical setting was compared in a subgroup of nosocomial strains. In these strains, based on MALDI-TOF spectra peaks, a clustering analysis was performed to identify changes in the microbial population over time, or under the selective pressure of the nosocomial environment.

## Materials and Methods

### Bacterial Isolates and Samples Collection

During the years 2015–2017, 445 strains of *Enterobacter* species have been isolated at the University Hospital Campus Bio-Medico of Rome, Italy. The species isolated and their relative number have been reported in **Table 1**, whereas the samples collected from the potential sites of infection has been described in **Table 2**. The study was performed using sample collected for the routine clinical diagnosis with the patient’s consent.

**Table 1:** *Enterobacter* species and their relative number isolated from the year 2015 to the year 2017.

Year	*E. cloacae*	*E. aerogenes*	*E. asburiae*	Total
	*n* (%)	*n* (%)	*n* (%)	*n* (%)
2015	84 (56)	60 (40)	7 (4)	151 (34)
2016	92 (62)	47 (32)	9 (6)	148 (33)
2017	105 (72)	26 (18)	15 (10)	146 (32)
Total *n* (%)	281 (63)	133 (30)	31 (7)	445 (100)


**Table 2:** Source of *Enterobacter* isolates during the years 2015–2017: type of samples collected.

Year	Blood culture	Respiratory sample	Ulcer or wounds	Nasal/rectal swabs	Urine	Total
	*n* (%)	*n* (%)	samples *n* (%)	*n* (%)	*n* (%)	*n* (%)
2015	20 (18)	11 (7)	68 (45)	16 (10)	36 (24)	151 (34)
2016	18 (12)	11 (7)	36 (24)	35 (24)	48 (32)	148 (33)
2017	18 (12)	7 (5)	64 (44)	18 (12)	39 (27)	146 (33)
Total *n* (%)	56 (12)	29 (6.5)	168 (38)	69 (15.5)	123 (28)	445 (100)


### Bacterial Identification

Bacterial identification was performed using the MALDI-TOF (Microflex LT, Bruker Daltonics, Germany) with the MALDI Biotyper 3.1 software version, and the MALDI-TOF VITEK MS-DS (bioMérieux, Marcy-l’Étoile, France), and the Saramis software.

### MALDI-TOF MS Biotyper (Bruker, Daltonics, Germany) Identification

Bacterial colonies were grown overnight on sheep blood agar and subjected to according to the MALDI Biotyper protocol (Bruker Daltonics GmbH, Bremen, Germany). Each isolate was smeared for ten times onto target slide (Bruker Daltonics GmbH, Bremen, Germany) (Angeletti et al., 2015).

Spectra were acquired by the standard recommended method using the Biotyper preprocessing standard method and the Biotyper Main-Spectrum (MSP) identification standard method (2,000 to 20,000 Da; linear positive method; laser frequency of 60 Hz). Species were identified using the MALDI Biotyper 3.1 and its standard database (Bruker Taxonomy database version 3.3.1). The software automatically acquired spectra and analyzed them by standard pattern matching against the spectra of the species used as reference. After comparing the unknown spectra comparison with the reference spectra, the log scores were reported. Values higher than 1.9 were considered reliable for the identification at the species level whereas values ranging from 1.9 to 1.7 were required for reliable identification at the genus level.

### MALDI-TOF VITEK MS v2.0 Identification (BioMérieux, Marcy-l’Étoile)

Strains subcultured on sheep blood agar plates (bioMérieux) at 37°C for 24 h were identified by the VITEK MS. Each isolate was smeared for six times onto the VITEK MS-DS target slide (bioMérieux), supplied in a 48-well microscope slide format, and divided into three acquisition groups of 16 spots each using a 1 μL disposable loop. The prepared samples were covered with 1 μL of α-cyano-4-hydroxycinnamic acid (CHCA) matrix solution (bioMérieux) and dried at room temperature. The mass spectra were acquired using a VITEK MS Axima Assurance mass spectrometer (bioMérieux). The isolates were identified using the Advanced Spectrum Classifier (ASC) algorithm, comparing the obtained spectra with the typical spectra of each organism in the VITEK MS 1.1 database (which includes more than 25,000 spectra covering 585 species). For system calibration and internal identification control, *E. coli* ATCC 8739 was used. The VITEK MS v2.0 system is equipped with Shimadzu Axima Assurance mass spectrometer linked to a reference database, known as Knowledge Base. During analysis, spectra within a range of 2,000 to 20,000 Da are recorded in linear positive mode at a laser frequency of 50 Hz and for each analysis, laser shots at different positions within the target well produce up to 100 mass profiles that are summed into a single, raw mass spectrum. The spectrum is then processed by baseline correction and peak detected. Data are used to query the database to determine the taxonomic identity in form of single species-level identification. The result from the first test with the VITEK MS, which provided a single choice at species level with ≥90% confidence, was used.

### Antimicrobial Susceptibility Test and Antimicrobial Activity

*Enterobacter* antimicrobial susceptibility tests were performed by Vitek2 Compact (bioMérieux, Marcy-l’Étoile, France) and the resistant phenotype further confirmed with the Kirby-Bauer method according to Clinical Laboratory Standard Institute (CLSI) and European Committee for Antimicrobial Susceptibility Test (EUCAST) (Gherardi et al., 2012).

The antimicrobial activity of the compounds recommended by EUCAST has been evaluated for the most frequently isolated species of *Enterobacter* (*E. cloacae* and *E. aerogenes*) by MIC_50_ and MIC_90_ determination, using GraphPad software^1^. The MIC_50_ and MIC_90_ have been calculated for 70 strains isolated in 2015 (44 *E. cloacae* and 26 *E. aerogenes*), for 62 strains in 2016 (45 *E. cloacae* and 17 *E. aerogenes*) and for 58 strains in 2017 (49 *E. cloacae* and 9 *E. aerogenes*).

### Clustering Analysis of MALDI-TOF MS Spectra

Spectra obtained for each isolate on MALDI-TOF MS Biotyper was loaded on ClinProTools by spectra grouping function, to allow grouping of all technical replicates in one biological replicate, named Class by the software. A Class dendrogram of all the study isolates was built using the ClinProTool dendrogram creation standard method (with the correlation distance measured by the average linkage algorithm) of the Biotyper 3.1 software (Bruker Daltonics, Germany). Clusters were consequently analyzed according to the arbitrary distance levels from 500 to 50.

Spectra obtained for each isolate on MALDI-TOF MS VITEK MS were compared each other by Saramis software analysis through which a hierarchical clustering of samples could be performed and the results represented as a dendrogram.

## Results

### Bacterial Isolates

Four hundreds and forty-five strains of *Enterobacter* species have been isolated at the University Hospital Campus Bio-Medico of Rome, Italy, from the year 2015 to the year 2017. The number of strains isolated in each year was constant ranging from 151 isolates in the year 2015 to 148 and 146 in the year 2016 and 2017, respectively (**Table 1**). The species isolated and their relative number and percentage have been reported in **Table 1**. Along the 3 years of the study, the most frequent species was *E. cloacae* (63%) followed by *E. aerogenes* (30%) and *E. asburiae* (7%). The number of *E. cloacae* remained constant during the years whereas a decrease of *E. aerogenes* and a gradual increase of *E. asburiae* was observed in the year 2017 (**Table 1**).

The type of samples collected from the potential sites of infection have been reported in **Table 2**. The most frequent samples of origin were ulcers or wounds (38%) followed by urine (28%) and surveillance nasal or rectal swabs (15.5%). *Enterobacter* species were isolated in blood cultures in 12% of cases.

### *Enterobacter* Identification by MALDI-TOF Platforms

*Enterobacter* strains were identified by two MALDI-TOF platforms and results compared, as reported in **Table 1**. The MALDI-TOF Biotyper system identified at the species level all isolate with a sensitivity of 100%. Strains were identified as *E. cloacae* in 281/445 (63%) of isolates, as *E. aerogenes* in 133/445 (30%) isolates and as *E. asburiae* in 31/445 (7%) isolates.

The MALDI-TOF VITEK MS system correctly identified *E. aerogenes* and *E. cloacae* isolates except in two cases (2 strains of *E. cloacae* were not identified), whereas failed to identify all *E. asburiae* strains. Globally the identification at the species level was not achieved in 33/445 isolates (7.5%) corresponding to a sensitivity of 92.5%.

### Antimicrobial Activity and Antimicrobial Susceptibility Test

**FIGURE 1:**
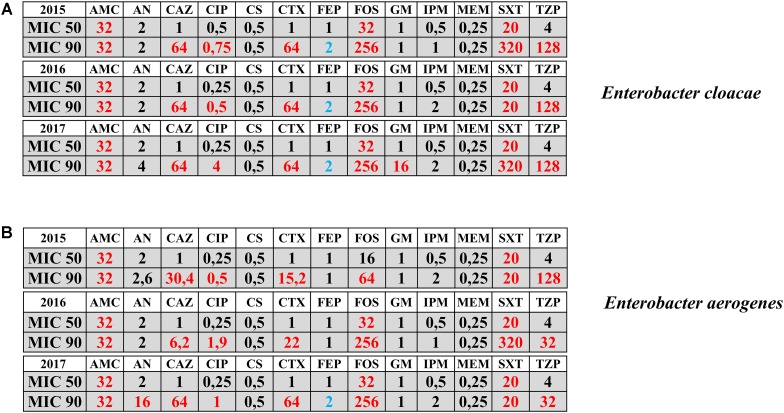
Antimicrobial activities in terms of MIC_50_ and MIC_90_ registered during the years 2015–2017 for *Enterobacter cloacae* (panel **A**) and *Enterobacter aerogenes* (panel **B**) isolates. Sensitive MIC (black color); Resistant MIC (red color); Intermedius MIC (blue color). AMC = amoxicillin-clavulanate; AN = Amikacin; CAZ = ceftazidime; CIP = ciprofloxacin; CS = colistin; CTX = Cefotaxime; FEP = Cefepime; FOS = Fosfomycin; GM = Gentamicin; IPM = Imipenem; MEM = meropenem; SXT = Trimethroprim/Sulfamethoxazole; TZP = piperacillin-tazobactam. Numbers indicate the MICs values.

The antimicrobial activities expressed in terms of MIC_50_ and MIC_90_ registered during the years 2015–2017 for *E. cloacae* and *E. aerogenes* have been represented in **Figure 1**. *Enterobacter* species are intrinsically resistant to ampicillin and cephalosporins of I and II generation. Regarding other antimicrobials, MIC_50_ was sensitive for both species except in case of Cefoxitin and trimethoprim/sulfamethoxazole. The MIC_90_ revealed the resistance for both species to cephalosporins of III and IV generation and piperacillin/tazobactam all over the 3 years of observation. Furthermore, the emergence of resistance to fluoroquinolones and aminoglycoside for both species was evidenced in the year 2017 (**Figure 1**).

Extended Spectrum Beta-Lactamase (ESBL) *Enterobacter* strains have been isolated along the 3 years of the study. A total of 131/445 (29%) isolates resulted ESBL, as reported in **Table 3**. The ESBL phenotype was identified in all three species of *Enterobacter* exactly in 79/281 (28%) *E. cloacae*, in 41/133 (31%) *E. aerogenes* and in 11/31 (35%) *E. asburiae* (**Table 3**). In the year 2017, 2/105 (1.9%) *E. cloacae* strains resulted MDR and carbapenem resistant. For these two strains, the carbapenem resistance was analysed by real-time PCR (GeneXpert Carba-R Assay, Cepheid^®^ Inc.) for the following *bla*KPC (KPC), *bla*NDM (NDM), *bla*VIM (VIM), *bla*OXA-48 (OXA-48), and *bla*IMP-1 (IMP-1) gene sequences. *bla*IMP-1 in one strain and *bla*VIM genes in the other, were detected.

**Table 3:** ESBL *Enterobacter* strains isolated from the year 2015 to the year 2017.

Year	ESBL *E. cloacae*	ESBL *E. aerogenes*	ESBL *E. asburiae*	Total
	strains *n*/total (%)	strains *n*/total (%)	strains *n*/total (%)	*n*/total (%)
2015	23/84 (27)	18/60 (30)	1/7 (4)	42/151 (28)
2016	20/92 (22)	16/47 (34)	2/9 (22)	38/148 (26)
2017	36/105 (34)	7/26 (27)	8/15 (53)	51/146 (35)
Total n/total (%)	79/281 (28)	41/133 (31)	11/31 (35)	131/445 (29)


### Clustering Analysis of MALDI-TOF MS Spectra

**FIGURE 2:**
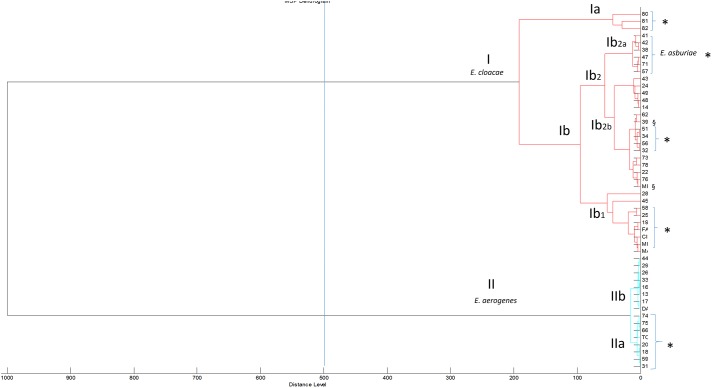
Class dendrogram of all *Enterobacter* isolates built by the MALDI-TOF Biotyper 3.1 software. Asterisks (^∗^) identify strains isolated in general surgery and geriatric wards; the symbol § identify multidrug-resistant (MDR) strains.

The class dendrogram of all *Enterobacter* isolates built by the ClinProTool dendrogram creation standard method using the Biotyper 3.1 software (Bruker Daltonics, Germany) revealed two major clusters (I,II) according to an arbitrary cut-off located at the distance level of 500 (**Figure 2**). In each cluster, distinct clades are evident. Cluster I, includes clades Ia and Ib further distinguished in the sub-clades I_b1_ and I_b2_. The sub-clade I_b2_ further includes the sub-clades Ib_2a_ and Ib_2b_. In cluster I all *E. cloacae* and *E. asburiae* strains are included, exactly *E. asburiae* isolates form a separate group represented in the sub-clade I_b2_, whereas all *E. cloacae* strains are distributed in the other clades and sub-clades of the cluster I (**Figure 2**). Cluster II includes all *E. aerogenes* strains distributed in two different clades (IIa and IIb). Within cluster I and II, strains isolated in patients admitted to general surgery and geriatric wards are located in the same clade or sub-clade separately from strains isolated in other hospital wards. In cluster I, the clade Ia includes only *E. cloacae* strains from general surgery and geriatric wards; in clade Ib general surgery and geriatric isolates are always grouped in the same sub-clades (Ib1,Ib2b) and in the sub-clade Ib2b are recovered the two MDR isolates. Interestingly, in the sub-clade Ib2a also *E. asburiae* strains are all from patients admitted in these two wards.

**FIGURE 3:**
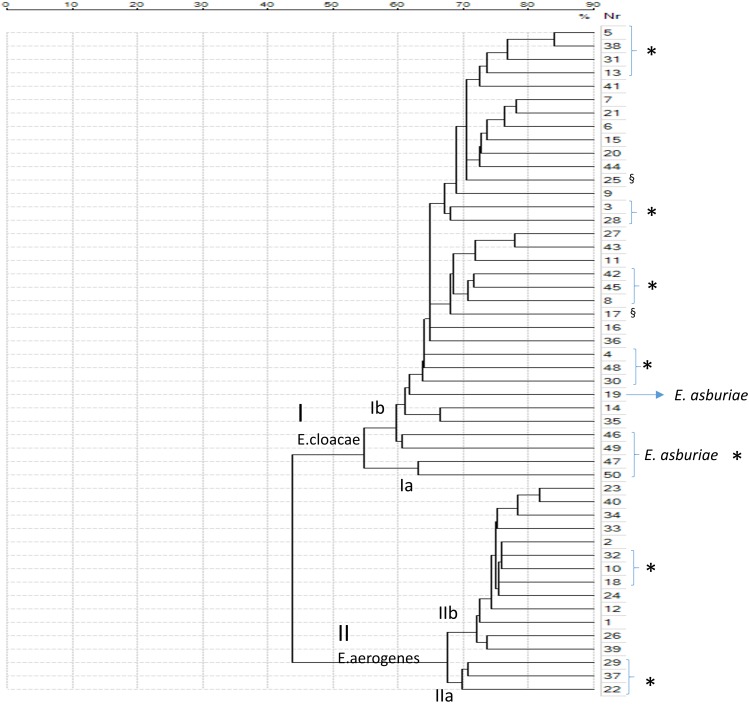
Class dendrogram of all *Enterobacter* isolates built by the MALDI-TOF VITEK MS Saramis software. Asterisks (^∗^) identify strains isolated in general surgery and geriatric wards; the symbol § identify multidrug-resistant (MDR) strains.

The class dendrogram of all *Enterobacter* isolates built by VITEK MS Saramis software, showed two major clusters I and II. Cluster I including *E. cloacae* strains and cluster II including *E. aerogenes* strains. In cluster I and cluster II, two different sub-clades are evident [Ia, Ib, IIa, and IIb (**Figure 3**)]. A distinct clade or sub-clade for *E. asburiae* strains is not represented, in fact these strains are identified as *Enterobacter* spp., even if these strains are located within the cluster I, distributed within the clades Ia and Ib including *E. cloacae* isolates.

In the clades Ia and Ib *E. cloacae* and *E. asburiae* isolates from general surgery and geriatric wards are included in the same clade or sub-clades separately from strains of others wards. In the clade II, *E. aerogenes* strains are distributed in two clades IIa and IIb, exactly strains isolated in general surgery and geriatric wards are represented in clade IIa whereas strains from other wards are grouped in clade IIb (**Figure 3**).

## Discussion

The study of the microbial ecology is fundamental in the era of antimicrobial resistance. Recently, the environment has been suggested as source and way of dissemination of resistance, recognizing to the environmental bacteria as well as to the different human microbiomes an important role (Martínez, 2008; Wright, 2010; Ashbolt et al., 2013; Finley et al., 2013; Pruden et al., 2013).

Resistant microorganism is rapidly evolving under the selective pressure exerted in the different environment where the microorganism is adapting.

The nosocomial setting represents a fertile environment where microorganism can rapidly evolve acquiring mobile genetic elements conferring antimicrobial resistance and where their spreading is the cause of fearsome infections.

The rapid identification of bacterial strains causing nosocomial infections, the knowledge of the resistance patterns and of the clonal dissemination pathway in the specific environment can represent an advantage especially in terms of morbidity, mortality and health costs savings.

In this study, the identification of *Enterobacter* pathogens circulating within the hospital setting and their antimicrobial susceptibility along 3 years of observation from 2015 to 2017 has been evaluated to have a picture of the circulation of these pathogens rapidly acquiring resistance to important antibiotics such as carbapenems. To gain deep knowledge about the circulation of these strains, a clustering analysis based on MALDI-TOF spectra was performed and the relationship existing between the isolates or any potential case of clonal transmission analyzed.

Based on the results obtained we could observe that the most frequent species isolated were *E. cloacae* and *E. aerogenes* with a gradual increase of *E. asburiae* in 2017, a species recently recognized as cause of severe infections (Brenner et al., 1986; Stewart and Quirk, 2001; Koth et al., 2012). The increasing circulation of the *E. asburiae* strains in the year 2017 in the nosocomial setting confirms the general increase observed in other settings and the potential enrollment as cause of clinically significant infections. Furthermore, the spreading of these emerging strains in the nosocomial setting predisposed to the rapid acquisition of antimicrobial resistance under the selective pressure of the antimicrobial compounds use. Noteworthy, to the consistent increase of *E. asburiae* strains corresponded an increase in the ESBL resistance phenotype that was evidenced in about 53% of the strains isolated in the year 2017.

The *Enterobacter* strains identification was based on MALDI-TOF platforms, recently introduced in the routine of the clinical microbiology laboratory for its rapidity and cost-effectiveness (Angeletti, 2017). The identification was simultaneously performed by the two most frequently used MALDI-TOF platforms and their performance compared. Both instruments showed a very good sensitivity and specificity except for *E. asburiae* identification that was reliable only using one of this, MALDI-TOF MS Biotyper. In fact in case of MALDI-TOF VITEK MS the two species *E. cloacae* and *E. asburiae* were grouped together and identified as a low discrimination result. This is in line with the observation that the ability of identification and discrimination between different species depends on the completeness of the database used for spectra matching after acquisition and its updating (Angeletti, 2017).

Regarding antimicrobial susceptibility expressed in term of MIC_50_ and MIC_90_ registered during the years 2015–2017, a raise of the MIC_90_ for cephalosporins of III and IV generation, piperacillin/tazobactam, fluoroquinolones and aminoglycoside was detected suggesting an increase of resistance for these antimicrobials under the selective pressure of the nosocomial environment. This was also confirmed by the isolation of ESBL strains for all three species, *E. cloacae*, *E. aerogenes*, and *E. asburiae* and by the emergence within the nosocomial setting in the year 2017 of *E. cloacae* MDR and carbapenem resistant strains. These data are in agreement with other studies suggesting the evidence of isolates carrying important determinants of carbapenem resistance such as *bla*IMP and *bla*VIM (Panopoulou et al., 2010; Sidjabat et al., 2015). These *Enterobacter* strains play an important role in nosocomial infections where carbapenems represent an important therapeutic option especially in case of systemic severe infections (Pottumarthy et al., 2003; Deshpande et al., 2006; Blanco et al., 2013). The rapid identification of these strains, the antimicrobial susceptibility and the resistant phenotype characterization together with the strict surveillance of the MDR strains are major concerns to limit the further spreading of these fearsome pathogens within the nosocomial setting.

The clustering dendrograms built on the basis of the spectra applying mathematics algorithm, showed two major clusters by both MALDI-TOF clustering analysis. These clusters included distinct clades or sub-clades where strains isolated from general surgery and geriatric wards were separated from strains of others wards. Interestingly, the clustering realized on the spectra similarity evidenced that those strains circulating in the general surgery and geriatrics wards where more strictly correlated than others isolated in different wards. A potential clonal route of transmission preferentially between these two wards could be suggested. This clonal route of transmission is in agreement with our previous studies performed in the same nosocomial setting but involving other gram-negative pathogens such as *K. pneumoniae* MDR strains (Angeletti et al., 2016; Cella et al., 2017). In these studies, gram-negative MDR strains causing nosocomial infections moved from the general surgery to the geriatric wards and, following this route, reached other patients in other hospital wards. The MALDI-TOF clustering applied to the *Enterobacter* spp. strains causing nosocomial infections within the same hospital setting confirmed the existence of this preferential way of transmission for gram-negative bacteria, probably as a consequence of the diagnostic and invasive procedure that are commonly used in these wards.

## Conclusion

The results of this study on *Enterobacter* pathogen, able to acquire determinant of resistance as non-metallocarbapenemase class A carbapenemase (Blanco et al., 2013; Chavda et al., 2016) as well as *K. pneumoniae*, suggest that the selective pressure existing in the nosocomial setting and the surgical or diagnostic procedure performed could influence the microbial ecology of the gram-negative pathogens. These findings represent an important source of information about the spreading of *Enterobacter*, an emergent pathogen for its ability to acquire determinants of antimicrobial resistance. Preventive measures based on active microbiological surveillance are needed to limit the dissemination of these microorganisms and guarantee the health status in the nosocomial environment.

## Author Contributions

LF, ER, MC, and SA conceived and designed the study. LF, AG, ED, SS, and MF collected the samples. LF, AG, ED, MF, EC, and SA performed the experiments. LF, ER, SS, MC, AS, AL, and GZ analyzed the data and wrote the paper. All authors read, reviewed, and approved the final manuscript.

## Conflict of Interest Statement

The authors declare that the research was conducted in the absence of any commercial or financial relationships that could be construed as a potential conflict of interest.

## Footnotes

1 https://www.graphpad.com/support/faq/fitting-bacterial-growth-data-to-determine-the-mic-and-nic/
